# The effect of N-acetylcysteine on inflammation and oxidative stress in cisplatin-induced nephrotoxicity: a rat model

**DOI:** 10.3906/sag-1903-225

**Published:** 2019-12-16

**Authors:** İnayet GÜNTÜRK, Cevat YAZICI, Kader KÖSE, Fatma DAĞLI, Bilal YÜCEL, Arzu YAY

**Affiliations:** 1 Department of Midwifery School of Health, Niğde Ömer Halisdemir University, Niğde Turkey; 2 Department of Biochemistry, Faculty of Medicine, Erciyes University, Kayseri Turkey; 3 Department of Chemistry, Çetin Şen Science and Art Center, Kayseri Turkey; 4 Department of Biochemistry, İzmir Konak Public Health Laboratory, İzmir Turkey; 5 Department of Histology and Embryology, Faculty of Medicine, Erciyes University, Kayseri Turkey

**Keywords:** Rats, cisplatin, oxidative stress, inflammation, N-acetylcysteine

## Abstract

**Background/Aim:**

Cisplatin is a highly effective chemotherapeutic agent used in the treatment of solid organ cancers. Besides its chemotherapeutic effectiveness, cisplatin administration is associated with numerous side effects. Of those, the most clinically significant and common effect is nephrotoxicity. Recent studies reported that oxidative stress and inflammation are probably the most important mechanisms that contribute to the nephrotoxicity. N-acetylcysteine (NAC) is an antioxidant and antiinflammatory agent. In the present study, the effects of NAC on cisplatin-induced nephrotoxicity were investigated.

**Materials and methods:**

Rats were divided into four groups each including eight rats: CONT, NAC-250, CP, and CP+NAC. Rats in experimental groups were treated intraperitoneally (i.p.) with a single dose of cisplatin (10 mg/kg body weight) and i.p. with NAC (250 mg/kg body weight) for three consecutive days. Nephrotoxicity was determined by plasma BUN and creatinine levels. In tissue samples, myeloperoxidase (MPO), nuclear factor-kappa B (NF-kB), high mobility group box-1 (HMGB-1), total oxidant status (TOS), and total antioxidant status (TAS) levels were measured. Kidneys were analyzed histopathologically as well.

**Results:**

It was revealed that cisplatin was not effective on MPO, HMGB-1 and NF-kB levels but did increase TOS levels and decrease TAS levels in tissue samples. Interestingly, NAC elevated MPO and HMGB-1 levels significantly. Nevertheless, NAC ameliorated histological and functional changes in kidney tissues.

**Conclusion:**

It is suggested that inflammation has a limited effect on cisplatin nephrotoxicity in this experimental design, and, as reflected by decreased BUN and creatinine levels, NAC can be used as an additional therapeutic agent in standard cisplatin treatment protocols.

## 1. Introduction

Cisplatin is one of the most effective chemotherapeutic agents used for the treatment of solid tumors. Despite its antitumor activity, it is also known for serious side effects. Major side effects include nephrotoxicity, neurotoxicity, and myelosuppression, and among these, the most serious and dose-limiting side effect that limits its clinical use and anticancer activity is nephrotoxicity. Despite its toxic characteristics, it is still widely used thanks to its therapeutic efficacy considering its risk/benefit ratio [1].

While cisplatin interacts with many cell components, the main biological target of the drug is deoxyribonucleic acid (DNA) [2]. However, as proximal tubule cells are nondividing cells, the nephrotoxic effect is believed to occur via mechanisms other than DNA damage. While different mechanisms are suggested for the development of nephrotoxicity, the most commonly accepted and investigated mechanisms are apoptosis, oxidative stress, and inflammation [3].

As no approved treatment protocol or specific antidote to be used against possible toxic effects of cisplatin use currently exists [4], different toxicity-preventing strategies are being rigorously investigated today.

Routine applications to prevent nephrotoxicity include creating forced diuresis using mannitol and furosemide, using hypertonic chlorine solutions, using alternative cisplatin regimens, avoiding combination with other nephrotoxic agents, and using it together with compounds thought to be able to prevent nephrotoxicity [5]. 

Despite intense prophylactic attempts, nephrotoxicity has been reported even in 20%–30% of patients who received a single dose of cisplatin [1]. As renal toxicity is accepted to be cumulative, it is suggested that successive use of cisplatin would increase the incidence and severity of toxicity [6]. However, despite the accepted opinion, platin levels in the renal tissues of patients who died during cisplatin treatment have been demonstrated to be based on the last treatment dose right before dying and not on the total dose the patient received [7]. Therefore, additional measures might be necessary to take precautions against the development of nephrotoxicity even in patients who use a single dose of cisplatin.

Being the acetylated precursor of L-cysteine, N-acetylcysteine (NAC) has been used for a long time as a mucolytic agent, and in conditions such as acetaminophen intoxication, doxorubicin-induced cardiotoxicity, stable angina pectoris, acute respiratory distress syndrome, and psychiatric disorders. Moreover, it has a very low toxicity level [8]. Being oxidized by various radicals and also acting as a nucleophile, NAC, thanks to these characteristics, can reduce the disulfide bridges in proteins, act as a free-radical capturer, and produce metal chelation [9]. Furthermore, NAC is also known for its antiinflammatory and antiapoptotic properties [10]. In addition to these direct effects, NAC also exerts its effect by increasing GSH levels [8,9]. 

This study investigated the contribution of oxidative stress and inflammation to cisplatin-induced nephrotoxicity, and also the association of NAC with these mechanisms and whether it is protective against nephrotoxicity in a rat model.

## 2. Materials and methods

The study was approved by the Erciyes University Local Ethics Committee for Animal Experiments (Ethics Committee No: 13/150). The experiments were performed in the Erciyes University Experimental Research and Application Center Laboratory.

### 2.1. Animals

Male Wistar albino rats (n = 32) 6–8 months old and weighing 280–445 g were housed under controlled standard environmental conditions of temperature (22 ±3 °C) and light (12/12-h light/dark cycle). Food and water were provided ad libitum. All the procedures were performed in compliance with the Helsinki Declaration and International Guiding Principles for Biomedical Research Involving Animals.

### 2.2. Experimental design

All rats planned to be used in the experiment were weighed and experimental groups were formed in such a way that the groups were not different from each other. After a 1-week acclimation period, animals were randomly divided into four groups (8 animals in each group): the control, cisplatin (CP), NAC (NAC-250), and cisplatin plus NAC (Cp+NAC) groups. The CP group was treated with a single intraperitoneal (i.p.) injection of 10 mg/kg rat body weight of cisplatin (Cisplatin DBL, 100 mg/100 mL), whereas the control group was injected with saline (1 mL/kg). The NAC-250 group was treated with i.p. administration of NAC (250 mg/kg; Asist, Bilim 300 mg/3 mL, 10%), while the Cp+NAC group was treated with a single dose of cisplatin (10 mg/kg, i.p.) and three consecutive days of NAC (250 mg/kg, i.p.) (Table 1). The animals in the last group were administrated NAC 4 hours after the cisplatin injection to avoid cisplatin–NAC interactions [11].

**Table 1:** Experimental design.

Group	n	Agent (i.p.)
CONT	8	1 mL/kg* saline
CP	8	10 mg/kg* cisplatin
NAC-250	8	250 mg/kg* N-acetylcysteine
CP+NAC	8	10 mg/kg* cisplatin + 4 h later and 2 consecutive days 250 mg/kg* N-acetylcysteine

*: Rat body weight; n: number of rats in each group; CONT: control group; CP: cisplatin group; NAC-250: N-acetylcysteine group; CP+NAC: cisplatin plus N-acetylcysteine group; i.p.: intraperitoneal.

Before and after the experiment, animals were housed separately in metabolic cages. For each animal, 24-h urine samples were collected and total volume was recorded. Samples from the collected urine were centrifuged for 15 min at 1000 × *g* and 4 °C and then kept frozen until analyzed for estimation of urea nitrogen, creatinine, and microalbumin. The body weight of each rat was recorded on the first and last days of the experimental period for calculation of change in body weight. 

At the end of the experiment, rats were anaesthetized with i.p. injection of 80 mg/kg ketamine (Ketalar flacon; Pfizer, USA) and 10 mg/kg xylazine (Rompun; Bayer, Germany). After dissection, blood samples were taken from the abdominal aorta and kidneys were rapidly excised and trimmed of connecting tissue. The left kidneys were washed free of blood with ice-cold 0.9% NaCl solution and they were then blotted and finally kept at –40 °C until use in biochemical analyses. For histological analysis, right kidneys were put immediately in 10% neutral formaldehyde.

### 2.3. Biochemical analysis 

Plasma and urine urea nitrogen and creatinine assays were performed photometrically on a Roche/Hitachi cobas c501 system (Roche Diagnostics, USA). 

For tissue analysis, weighed kidney samples were homogenized in ice with 0.015 M phosphate buffer (pH 7.5) in a ratio of 1:4. They were then centrifuged at 4 °C and 20,000 × g for 15 min. The supernatant was used for total oxidant capacity (TOC), total antioxidant capacity (TAC), myeloperoxidase (MPO), high mobility group box-1 (HMGB-1), and nuclear factor kappa B (NF-kB) analysis. 

TOS (RL0024) and TAS (RL0017) were evaluated photometrically in rat renal tissues supernatants with available commercial assay kits (Rel Assay Diagnostics, Turkey).

In rat kidney tissues, MPO (Cat No: 201-11-0575), NF-kB (Cat No: 201-11-0288), and HMGB-1 (Cat No: 201-11-0258) levels were measured according to the manufacturer’s instructions using commercially available ELISA kits (Sunred Biological Technology Co., China).

Tissue biochemical parameter results were obtained by proportioning to protein values calculated by the Lowry method [12] in the same tissues.

### 2.4. Histological evaluation

For light microscopic evaluations, the kidney samples were fixed in 10% buffered formalin for 48 h and processed for routine paraffin embedding. For general morphological evaluation, approximately 5-µm-thick sections were stained with hematoxylin and eosin (H&E). All of the stained sections were observed and photographed with a digital camera (Olympus C-5060; Olympus, Japan) attached to a photomicroscope (Olympus BX51; Olympus, Japan).

The microscopic scoring of the right kidneys was carried out in a blinded fashion by a histologist who was unaware of the treatment groups and assigned a score that represented the approximate extent of hemorrhage, tubular epithelial cell damage, and necrosis according to Abdelrahman et al. [13]. These parameters were evaluated on a scale of 0–3, which ranged from absent (0) and mild (1) to moderate (2) and severe (3) (Table 2). 

**Table 2:** Histological scoring table.

	Absent	Mild	Moderate	Severe
Hemorrhage	0	1	2	3
Tubular epithelial cell damage	0	1	2	3
Necrosis	0	1	2	3

### 2.5. Statistical analysis 

Statistical analysis of the study data was performed with IBM SPSS Statistics version 23 software. Conformity of the variables to normal distribution was assessed with the Shapiro–Wilks test. Statistical data were stated as mean ± standard deviation (SD) or median and interquartile range for normally and nonnormally distributed variables, respectively. A paired t-test was used to compare two dependent groups. One-way analysis of variance (ANOVA) was used to assess the effect of NAC treatment on biochemical parameters, urine volume, and rat body weight. If statistically significant differences were found for the groups, the data were further analyzed using the Tukey multiple comparison test. The differences among the groups were evaluated by using the Kruskal–Wallis test for histological parameters and the comparisons between every two groups were made using the Mann–Whitney U test. Data were considered statistically significant at P < 0.05. 

## 3. Results

While there was no difference between the CONT and NAC-250 groups in terms of poststudy plasma BUN and creatinine levels, a significant increase was present in the CP and CP+NAC groups compared to the CONT and NAC-250 groups. Again, in terms of those two parameters, a significant decrease was observed in CP+NAC group compared to the CP group (Table 3).

As seen in Table 4, no significant difference was observed between the prestudy urine volumes of the groups. When intragroup pre- and poststudy values were compared, an increase was observed in the urine volumes of all groups except for NAC-250. In the intergroup comparison at the end of the study, urine volume was only increased in the CP+NAC group compared to the control group; no difference was observed in the CP and CP+NAC groups, and urine volume was significantly higher in the CP and CP+NAC groups compared to the NAC-250 group. For microalbumin levels, no difference was observed between the groups in terms of both pre- and poststudy values, and in intragroup comparisons, a significant increase was observed in the CP group compared to the prestudy value (Table 4).

**Table 3:** Poststudy BUN and plasma creatinine levels of the rats.

	Groups				
	CONT (n = 8)	NAC-250 (n = 8)	CP (n = 8)	CP+NAC (n = 8)	P
	Mean ± SD				
BUN (mg/dL)	18.77 ±1.90	16.09± 2.10	137.09 ± 33.82	102.4±4.67	0.001
Creatinine (mg/dL)	0.44 ± 0.08	0.38 ± 0.04	3.59 ± 1.01	1.87 ± 0.20	0.001

P: P-value for the measurements after the experiments among the four groups.

**Table 4:** Pre- and poststudy 24-h urine volumes and microalbumin levels of the rats.

	Groups				
	CONT (n = 8)	NAC-250 (n = 8)	CP (n = 8)	CP+NAC (n = 8)	Significance
	Mean ± SD				
Urine volume (mL) Before After	6.17 ± 2.05 9.89 ± 3.17	8.24 ± 2.087.3 7 ± 1.23	6.79 ± 2.89 14.34 ± 5.43	6.65 ± 1.70 20.47 ± 6.84	P_1_: 0.301 P_2_: 0.001
P*	0.028	0.238	0.002	0.001	
Microalbumin (mg/dL) Before After	3.47 ± 2.08 3.80 ± 2.08	2.98 ± 1.69 5.00 ± 2.68	1.87 ± 1.56 5.97 ± 4.50	2.97 ± 1.60 5.59 ± 3.73	P_1_: 0.330 P_2_: 0.602
P*	0.675	0.053	0.040	0.104	

P1: P-value for basal measurements among the four groups. P2: P-value for the measurements after experiments among the four groups. P*: P-value for before and after measurements for each group.

No difference was observed for the prestudy body weights of the rats; however, a statistically significant decrease was observed in all groups at the end of the study. While no significant difference was observed between the groups at the end of the study, when percentage changes were compared between the groups, the weight loss that was not observed in the control group and NAC group was observed in both groups receiving cisplatin (CP and CP+NAC groups). On the other hand, no difference was observed for % change in the CP+NAC group compared to the CP group (Table 5). 

**Table 5:** Change in the body weights of the rats in the study group.

	Groups				
	CONT (n = 8)	NAC-250 (n = 8)	CP (n = 8)	CP+NAC (n = 8)	P
	Mean ± SD				
Rat body weights (g) Before After % Change	363.14 ± 60.98 353.5 ± 54.81 2.45 ± 1.72	353.63 ± 24.38 348.0 ± 21.29 1.54 ± 1.35	357.54 ± 28.24 308.97 ± 21.24 13.44 ± 3.88	370.69 ± 51.08 317.55 ± 43.69 14.32 ± 1.75	0.877 0.067 0.001
P*	0.007	0.018	0.001	0.001	

P: P-value among the four groups. P*: P-value for before and after measurements for each group.

Tissue MPO and HMGB-1 values were significantly increased only in the NAC-250 group compared to the CONT group and there was a numerical increase in NF-kB levels. Compared with the NAC-250 group, a significant decrease was observed in the MPO, HMGB-1, NF-kB, and TAS levels of the CP and CP+NAC groups; no significant difference was observed between the groups that received cisplatin alone or combined with NAC. TOS levels were observed to increase with cisplatin use; however, they could not be decreased by NAC administration (Table 6). 

**Table 6:** Changes in the oxidative stress and inflammatory parameters of the rats in the study groups.

	Groups				
	CONT (n = 8)	NAC-250 (n = 8)	CP (n = 8)	CP+NAC (n = 8)	P
	Mean ± SD				
MPO (ng/mg protein)	1.12 ± 0.29	1.64 ± 0.32	0.82 ± 0.20	0.84 ± 0.18	0.001
NF-κB (ng/mg protein)	0.46 ± 0.05	0.56 ± 0.09	0.35 ± 0.05	0.40 ± 0.14	0.001
HMGB-1 (ng/mg protein)	0.58 ± 0.16	0.82 ± 0.20	0.44 ± 0.11	0.47 ± 0.06	0.001
TAS ((mmol Trolox equiv./g protein)	0.024 ± 0.003	0.025 ± 0.003	0.017 ± 0.002	0.016 ± 0.003	0.001
TOS (µmol H_2_O_2_ equiv./g protein)	0.112 ± 0.012	0.122 ± 0.015	0.133 ± 0.016	0.136 ± 0.018	0.016

P: P-value among the four groups.

When renal tissue was assessed for hemorrhage, tubule cell damage, and tubular necrosis, no difference was observed between the CONT and NAC-250 groups and a significant increase was observed in the CP group compared to the CONT and NAC-250 groups. Hemorrhage and necrosis improvement was observed in the CP+NAC group but there was no difference compared to the control group. When assessed for tubule cell damage, while there was still difference compared to the CONT and NAC-250 groups, significant improvement was observed in the CP+NAC group compared to the CP group (Table 7).

**Table 7:** Histological study results.

	Groups				
	CONT (n=8)	NAC-250(n = 8)	CP (n = 8)	CP+NAC(n = 8)	P
	Median (interquartile range)				
Hemorrhage	0.0 (0.0–1.0)	0.5 (0.0–1.0)	3.0 (2.25–3.0)	1.5 (0.0–2.0)	0.001
Tubule epithelial cell damage	0.0 (0.0–1.0)	1.0 (0.25–0.1)	3.0 (2.0–3.0)	2.0 (2.0–2.0)	0.001
Tubular necrosis	0.0 (0.0–0.0)	0.0 (0.0–1.0)	2.0 (2.0–2.75)	0.0 (0.0–1.0)	0.001

P: P-value among the four groups.

Histopathologically, the saline-administrated rats had normal kidney architecture. In the CP group, the kidneys were generally worn and had a morphological appearance that had lost its natural color and showed significant hemorrhage, necrosis, and tubule cell damage. Vacuolization of tubular cells and brushed edge loss in proximal tubules were observed. The NAC-250 group had very mild hemorrhage, necrosis, and tubule cell damage. In the CP+NAC group, hemorrhage and tubular epithelial cell damage were observed similarly to the CP group, but cells with necrosis were fewer in number (Figure). 

**Figure 1:**
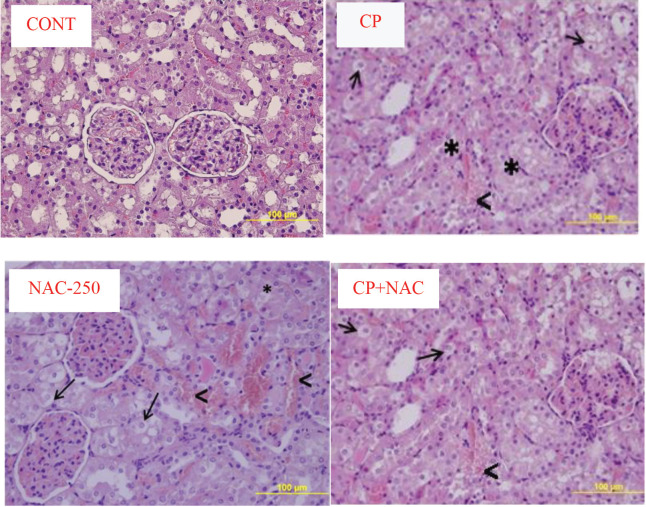
Morphology of the kidney tissue. Images are representative of the H&E-stained sections of kidneys from the experimental groups. CONT: Kidney tissue showing normal structure in control group; CP: cisplatin-injured kidney tissue obtained from the cisplatin group; NAC-250: NAC-treated group; CP+NAC: kidney tissue obtained from the cisplatin + NAC treated group. Original magnification: 40×, scale bar: 100 μm. Thick arrow (→): hemorrhage; arrowhead (<): interstitial edema; star (*): vacuolization.

## 4. Discussion

Cisplatin is one of the most potent chemotherapeutic agents commonly used for the treatment of cancer [4]. Clinical use of cisplatin leads to successful results in the treatment of many solid organ tumors including those of the head, neck, lungs, testes, and ovaries [14]. However, cisplatin and its derivatives also have side effects limiting their use, including mainly nephrotoxicity, followed by ototoxicity, cardiotoxicity, and myelosuppression [4].

Various agents with antioxidant properties such as selenium, vitamin E, dimethyl thiourea (DMTU), theophylline, amifostine, and NAC were used as chemoprotectives for the prevention of cisplatin-induced nephrotoxicity, and positive effects have been demonstrated [15]. The only agent approved by the US Food and Drug Administration for the prevention of cisplatin nephrotoxicity in non-small-cell lung cancer and advanced ovarian cancer is amifostine [16]. However, the high cost, side effects, and negative effects on the antitumor activity of cisplatin limit the use of amifostine [17]. In addition to its chemical similarity to amifostine, as an agent that is being used in clinical practice for many years and is more cost-effective without serious side effects, NAC [18] was chosen for this study.

The possibility of negative effects on anticancer efficacy of the chemotherapeutic agent in use is one of the major factors limiting the clinical use of a chemoprotective agent [19]. Different studies have shown the advantages of the timing and/or the route of the administration to minimize the interaction between the chemotherapeutic agent in use and the chemoprotective agent, and maximize the benefits of the chemoprotective agent [11,20,21].

Showing the nephroprotective effects obtained by NAC on tumor chemotherapy together, Muldoon et al. showed that NAC administered 4 h after cisplatin administration had no negative effect on antitumoral activity [11]. Therefore, an interval of 4 h was observed between the administrations of two agents.

The effects of NAC on cisplatin-induced renal toxicity were evaluated via different pathways in the literature. It has been demonstrated that it is possible to improve the demonstrated parameters of cisplatin-induced weight loss [11,13,22], apoptosis induction and caspase activation [20,23], oxidative stress and inflammation [23,24], and renal hemodynamic changes such as renal blood flow and blood pressure [13] by NAC. Cisplatin-induced acute renal injury can be described as acute renal failure characterized by decreased renal blood flow, which also aggravates the GFR decrease. As this hypofiltration is usually closely associated with minimal tubular proteinuria and elevated plasma BUN/ creatinine levels [1], these are the parameters that are usually evaluated in studies on cisplatin nephrotoxicity. 

In this study, findings of significant hemorrhage, necrosis, and tubule epithelial cell damage (vacuolization in tubule cells, loss of brush border in proximal tubules), which had a direct positive association with low urine and high plasma BUN and creatinine levels in rats that received cisplatin, proved the development of nephrotoxicity in rats receiving cisplatin. Moreover, proteinuria, which was assessed using microalbumin levels in this study, is believed to be an important indicator of nephropathy. Glomerular damage, which is especially seen at higher doses, is implicated for the development of proteinuria [14]. 

Consistent with this study, it has been suggested that cisplatin administration combined with NAC at different doses and times can decrease the levels of serum/plasma BUN and creatinine levels [13,21,22,24]. In addition to the demonstrated functional improvement, histopathological improvement was also observed in this study and different studies in the literature [13,24,25]. Even though the rat species, the dose, and the method and timing of administration of cisplatin and NAC were different in those studies, comparable results were obtained, and this is striking. 

However, while BUN and creatinine levels were significantly decreased compared to the CP group in this study, they did not reach the levels of the control group. The timing of NAC administration after cisplatin is also believed to be a factor in this situation. Likewise, Muldoon et al. also obtained comparable results with comparable timing of administration [11].

The most common gastrointestinal side effects of platin-containing chemotherapeutics such as cisplatin include nausea, vomiting, diarrhea, constipation, and oral mucositis. These side effects lead to rapid weight loss secondary to malnutrition and dehydration in patients [26]. While there are some studies in the literature showing that there is no change in body weight in rats receiving cisplatin [27,28], other studies generally reported decrease in body weight [11,13,21,22]. Significant weight loss was observed in all groups in this study whether receiving or not receiving cisplatin. This might be explained by rats staying in metabolic cages for a total of 2 days at the beginning and end of the experiment and an impaired diet due to this stay. In addition to this, weight loss observed during the experiment in the groups receiving cisplatin was determined to be approximately 14%, which was considerable compared to the other groups. Metabolic cage-induced lack of nutrition, which was common for all groups, together with gastrointestinal toxicity-associated modest diarrhea and tubular damage-induced severe polyuria might explain the weight loss in rats receiving cisplatin. However, contrary to the changes in biochemical parameters, as the excessive weight loss observed with cisplatin administration was close to the level observed in the CP+NAC group, it can be said that at this dose and timing, NAC cannot prevent the gastrointestinal toxicity of cisplatin, which is believed to cause weight loss.

One of the outcomes of cisplatin-induced renal tubular damage is severe polyuria [3]. While polyuria was shown in this study and also in different studies in the literature [29,30], there are also studies reporting the opposite [13,31]. Abdelrahman et al. [13] reported that urine output remained unchanged with cisplatin administration and associated this entity with diarrhea-induced excessive fluid loss and 95% decrease in CCr. 

While it was shown that NAC does not have a polyuric effect when the results of the NAC-alone group were evaluated, a surprising increase in the 24-h urine volume was observed in CP+NAC group compared to all other groups, including the CP group. Similarly, Abdelrahman et al. [13], who administered cisplatin and NAC together, demonstrated a substantial numerical increase in their CP+NAC group compared to the cisplatin group, though the difference was not statistically significant. However, this phenomenon could not be explained. 

The etiology of cisplatin nephrotoxicity involves many complicated and unclear factors and various signal pathways. However, the main ones especially include SOR production and antioxidant system dysfunction [24], and, accepted as an extension of this, the inflammatory response mediated by NF-kB, a prototypical proinflammatory agent [19].

In support of the contribution of free radicals to cisplatin-induced acute renal failure, Matsushima et al. [32] demonstrated that DMTU and sodium benzoate, which play a role as free-radical capturers, prevent cisplatin-induced malondialdehyde (MDA) increase, reduce the elevation in serum creatinine levels and NAG excretion and thereby tubular damage formation, and increase the regeneration of renal tubular cells. Moreover, Shalby et al. [24] reported reduced total antioxidant capacity in addition to high plasma/tissue MDA and NO levels due to antioxidant system failure in rats receiving cisplatin. This study showed that TOS levels in the renal tissues were significantly increased and TAS levels were decreased compared to the controls. On the other hand, NAC administered together with cisplatin did not have any significant effect on the cisplatin-induced TOS increase and TAS decrease in the renal tissues. In this case, it can be said that NAC is not used for its direct radical capture effect, but rather for its beneficial effect of increasing GSH concentrations, which was shown to be more effective in in vivo conditions [9], which was demonstrated in this study by the BUN and creatinine levels. However, the lack of the expected increase in TAS levels might be due to molecular interaction of high-dose cisplatin with GSH [33]. 

As it is known that cisplatin nephrotoxicity also involves inflammation in addition to oxidative stress [16], it can be said that the assessment of activity and/or concentration of neutrophil-specific MPO would be important for the diagnosis and monitorization of nephrotoxicity. Moreover, it was demonstrated that MPO levels were significantly higher, or in other words, neutrophil activation was increased, in the animals receiving cisplatin compared to the healthy animals [27,34]. 

However, a numerical decrease was observed in the MPO levels as a result of cisplatin administration in this study, though this was not statistically significant. While neutrophil infiltration-mediated inflammation is considered to be one of the possible causes of cisplatin-induced nephrotoxicity, no improvement was reported in the tubular necrosis and renal functions in mice with cisplatin-induced acute renal failure, which received the RB6-8C5 monoclonal antibody to create neutrophil depletion in peripheral and renal tissues, even though there was no neutrophil infiltration [35]. In other words, it can be said that the inflammatory status caused by severe cisplatin-induced renal toxicity as demonstrated by renal functions and histological examination is not caused by neutrophils.

Moreover, the fact that the inhibitory effect of cisplatin on neutrophil activation has been demonstrated [36] and renal platin accumulation has been demonstrated in different studies in the literature [13,31], even though plasma/renal cisplatin levels were not assessed in the present study, might suggest that the numerical decrease in tissue MPO values might be due to the inhibitory effect of cisplatin on neutrophil activation as a result of renal tissue accumulation. 

No study was found in the literature showing the effect of NAC in combination with cisplatin on neutrophil activation and/or MPO levels. Furthermore, MPO, NADPH oxidase, and NF-kB p65 subunit activities in the renal tissues of the rats receiving cisplatin together with fisetin, which is a natural antioxidant and was shown to increase GSH levels, were lower than in the animals receiving cisplatin alone [37]. The present study did not find any significant change in the renal tissue MPO values in the CP and CP+NAC groups compared to the CONT group. 

Overall, there was no study found in the literature showing that NAC alone has a direct effect on MPO levels, but increased MPO values were observed after NAC administration in the present study. The present study also showed that neutrophil activation, and thus tissue MPO levels, which are believed to be suppressed by cisplatin administration, remain unchanged by NAC administered together with cisplatin. 

This study detected a numerical but statistically nonsignificant decrease in the tissue NF-kB levels of the animals receiving cisplatin. These results seem to be consistent with the results of similar studies in the literature [38,39]. This might be because of the changes in the animal species used, cisplatin dose, and/or the timing of euthanasia. That being said, the decreased NF-kB levels in this study might be explained by the fact that cisplatin causes NF-kB inhibition by binding the “kB” regions, which are the NF-kB DNA binding regions, and, as a result, leads to an increase in the apoptosis and necrosis sensitivity of the tumor cells [40]. In the end, it can be thought that early NF-kB increase due to tissue damage during cisplatin treatment may cause negative feedback as it is not possible to bind to the “cisplatin-blocked kB” regions on the DNA and thereby reduce its own level by the time of euthanasia. Additionally, in another study, it was specified that NF-kB, which was initially activated by SOR under modest conditions, might be inhibited by SOR to sustain vitality under severe conditions triggering cell death [41]. 

Examining the effects of NAC on the DNA-binding activity of NF-kB in different cell lines, Liu et al. [42] showed that NAC induces cytoplasmic inhibitor of nuclear factor kappa-B kinase- and phosphatidylinositol 3 kinase-mediated p65 subunit phosphorylation and NF-kB activation in a dose-dependent manner. As they did not obtain the same results with different antioxidants such as vitamin C or tetramethyl thiourea, they suggested that this effect does not depend on the antioxidant characteristic of NAC. According to these results, it is possible to say that numerically increased NF-kB levels in rats that received NAC alone in this study might be because of the NAC-mediated NF-kB activation. Similarly, the increase in MPO might also be associated with this pathway, and the significant increase observed in MPO levels of the NAC-250 group might be due to induction of NOX-2 activation by NF-kB [41]. 

It has also been reported that the inflammatory effects of cisplatin might also be mediated by the damage-associated molecular pattern (DAMP), which develops secondary to intracellular damage and causes chemokine and cytokine release [16]. Therefore, as a well-known member of the DAMP family and also known for contributing to sterile inflammation, HMGB-1 [43] might be contributing to the toxic status seen during cisplatin treatment. 

However, there is no generally accepted correlation between HMGB-1 and cisplatin cytotoxicity [44]. Park et al. [45] suggested that poly(ADP-ribose) polymerase 1-dependent HMGB-1 release increases due to oxidative stress seen in the renal proximal tubule cells after high-dose cisplatin administration, and this contributes to the cisplatin-induced inflammation. While the presence of oxidative stress was demonstrated by increased TOS and decreased TAS levels in the present study, such an increase was not observed in HMGB-1 levels.

The present study found that HMGB-1 concentration in the renal tissue was increased in the NAC-250 group compared to the control, and it was significantly higher in the cisplatin groups (CP and CP+NAC) than the NAC-250 group and numerically lower than in the control group. However, no study was found in the literature on the effect of NAC against HMGB-1 levels changing after cisplatin administration.

That being said, there are studies showing that HMGB-1 increase can be reversed by NAC expression in support of this increase being mediated by oxidative stress [46,47]. Yang et al. [47] demonstrated that HMGB-1 increase due to SOR induced by high glucose concentrations in the human proximal tubule epithelial cell line can be reversed by NAC. 

However, the fact that the significant increases in tissue MPO and HMGB-1 levels in the NAC-250 group accompany damage that was demonstrated histopathologically but not biochemically suggests that NAC causes inflammation-mediated tissue damage, albeit limited. Known to have very minimal toxic effects, NAC was not studied in this study for this effect. Future studies are needed to explain this phenomenon.

While the changes in the urine/blood urea nitrogen and creatinine levels seen with NAC administration after cisplatin did not reach the levels of the control group, certain histopathological and biochemical improvement was observed. However, a satisfactory assessment of the inflammatory status could not be performed due to possible interactions between cisplatin and the parameters believed to reflect the extent of the inflammation in the renal tissue. Nonetheless, when the results of this study are assessed histologically and in terms of renal function tests, it would be more appropriate to use NAC for long-term treatments at low doses to reduce the cisplatin-induced tissue damage.

## Acknowledgment

This study was supported by the Research Fund of Erciyes University (TDK.2014-5056).
