# Erratum to “The effect of N-acetylcysteine on inflammation and oxidative stress in cisplatin-induced nephrotoxicity: a rat model” [Turkish Journal of Medical Sciences 49 6 1789 1799]

**DOI:** 10.55730/1300-0144.6057

**Published:** 2025-09-01

**Authors:** İnayet GÜNTÜRK, Cevat YAZICI, Kader KÖSE, Fatma DAĞLI, Bilal YÜCEL, Arzu YAY

**Affiliations:** 1 Department of Midwifery School of Health, Niğde Ömer Halisdemir University, Niğde, Turkey; 2 Department of Biochemistry, Faculty of Medicine, Erciyes University, Kayseri, Turkey; 3 Department of Chemistry, Çetin Şen Science and Art Center, Kayseri, Turkey; 4 Department of Biochemistry, İzmir Konak Public Health Laboratory, İzmir, Turkey; 5 Department of Histology and Embryology, Faculty of Medicine, Erciyes University, Kayseri, Turkey

Due to an inadvertent error during the labeling of images in the figure included in the article, the authors have provided a revised version of the figure. In this updated version, the image corresponding to the NAC-250 group has been replaced with a new photograph, and all labels have been corrected accordingly.

This correction does not affect the conceptual content, results, statistical analysis, or conclusions of the article.

The authors apologize for any confusion this may have caused.

**Figure:**
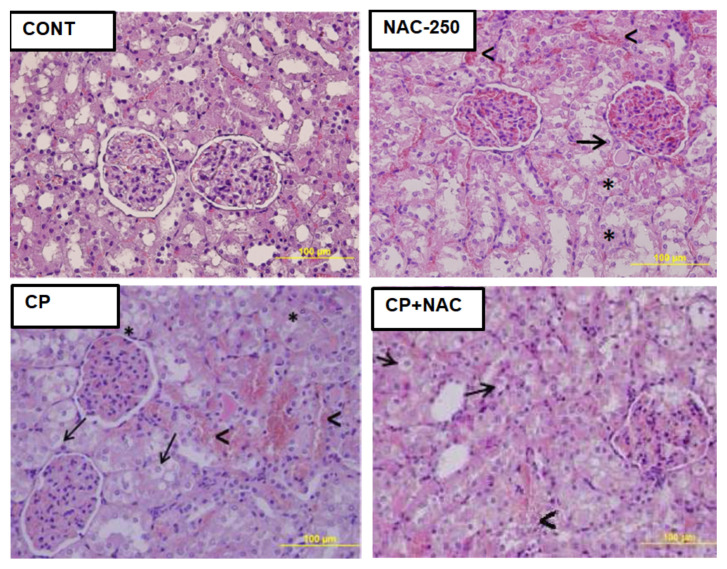
Morphology of the kidney tissue. Images are representative of the H&E-stained sections of kidneys from the experimental groups. CONT: Kidney tissue showing normal structure in control group; CP: Cisplatin-injured kidney tissue obtained from the cisplatin group; NAC-250: NAC-treated group; CP+NAC: Kidney tissue obtained from the cisplatin + NAC treated group. Original magnification: 40×, scale bar: 100 μm. Thick arrow (→): vacuolization; arrowhead (**<**): hemorrhage; star (*): necrosis.

